# How Adherence to an Evidence-Based Targeted Intervention Procedure is Related to Intervention Effectiveness?

**DOI:** 10.1007/s11121-026-01917-z

**Published:** 2026-04-21

**Authors:** Eerika Johander, Lydia Laninga-Wijnen, Daniel Graf, Daniela V. Chávez, Christina Salmivalli

**Affiliations:** INVEST Research Flagship Center/Department of Psychology and Speech-Language Pathology, University of Turku, 20014 Turku, Finland

**Keywords:** Targeted interventions, Victimization, Bullying, Implementation fidelity

## Abstract

Research suggests that although teachers’ targeted interventions can stop bullying, they still fail in about one-fourth of cases. Yet, most studies to date have not considered how targeted interventions were implemented, leaving open the possibility that improper implementation contributed to these failures. To address this gap, we examined the extent to which school personnel implementing the KiVa® antibullying program in Finland adhered to the program-recommended targeted intervention procedure when addressing bullying cases, and whether modifications to the procedure, influenced intervention effectiveness. We further tested the specific effects of two types of modifications – adaptations and omissions – on effectiveness. Data were collected using ecological momentary assessment, with school personnel documenting in a mobile application the steps they took when addressing bullying cases. The sample included 341 cases involving 396 victimized students (53% female, *M*age = 12.39 SD = 2.08) and 733 bullying students (13% female, *M*age = 12.52 SD = 1.96) from 22 primary and secondary schools. The results indicated that adherence to procedure varied considerably across intervention steps, and adherence to the full procedure was low. Interventions were, however, more effective when school personnel adhered to the procedure than when they made modifications. Moreover, interventions were least effective, when steps were omitted, whereas adaptations did not significantly reduce effectiveness compared to full adherence, though the trend was in the same direction as with omissions. These findings suggest that closer adherence to evidence-based procedures tends to lead to better outcomes in targeted bullying interventions.

## Introduction

School bullying, defined as repeated peer aggression involving a power imbalance (Olweus, 1993), remains a global challenge in education (Ariani, et al., 2025). Therefore, providing teachers with evidence-based guidelines for intervening in bullying cases is essential. Many school-based anti-bullying programs combine universal preventive components directed to all students (e.g., student lessons) with guidelines for targeted interventions addressing specific bullying cases (e.g., discussions with students involved). Program evaluations typically estimate changes in victimization and bullying rates over time for entire anti-bullying programs in intervention schools compared with control schools (Gaffney et al., 2019), whereas only a few studies have specifically examined the effectiveness of targeted interventions in stopping ongoing bullying targeted at particular children. These studies suggest that although teachers’ targeted interventions often stop bullying, they still fail (i.e., the bullying continues) in 20–30% of cases, even when evidence-based guidelines are provided (e.g., Garandeau et al., 2014; Johander et al., 2024). While this percentage may seem discouraging, these studies did not examine whether interventions were implemented as intended, leaving open the possibility that insufficient fidelity to targeted intervention protocols contributed to their limited effectiveness.

**Fig. 1:**
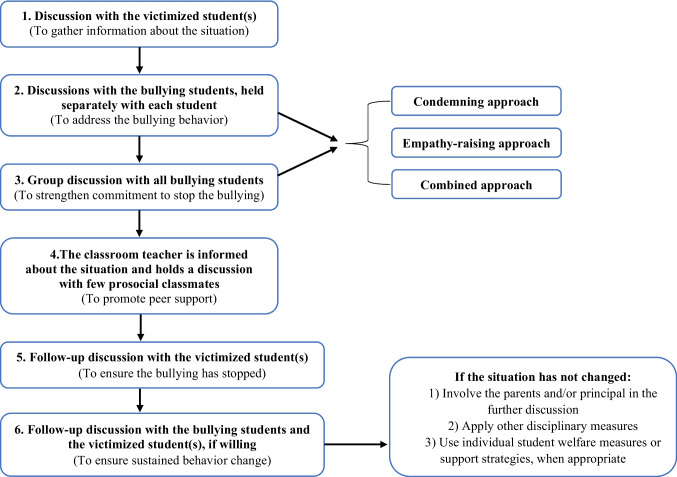
Steps in the targeted intervention procedure. The purpose of each step is provided in parentheses

Implementation fidelity refers to the degree to which a program or intervention is delivered as intended, and higher fidelity has been consistently linked to better outcomes (Durlak & DuPre, 2008). Yet, in practice, the implementation of school-based programs varies (Goncy et al., 2015). Teachers may face, for example, organizational (e.g., limited resources) or contextual (e.g., competing school initiatives) barriers, and describe a need for flexibility to fit program into everyday school life (Herkama et al., 2022). There is a clear call for research examining which components of bullying prevention programs can be modified and which require adherence to program guidelines to retain effectiveness (Herkama et al., 2022; Tolmatcheff & Veenstra, 2026). In anti-bullying research, implementation studies have mainly focused on lesson delivery (e.g., Haataja et al., 2014), while the implementation of other program components remains largely unexplored. To date, only one study, conducted in the context of KiVa anti-bullying program in Finland, has examined the implementation of targeted interventions (Johander et al., 2021). Although nearly 70% of schools initially adhered to the guidelines using evidence-based intervention approaches, over the years many shifted to their own adaptations, resulting in weaker effects. That study, however, focused only on the overarching approach chosen (i.e., program-recommended vs. own adaptation), rather than whether the specific steps of the program-recommended intervention procedure (Fig. 1) were followed. Also, it relied on teachers’ annual reports of intervention practices, which may be prone to recall bias and imprecision, as implementation can vary across cases that were handled during the school year. To address these limitations, we used ecological momentary assessments (collecting reports of each intervention step when they were implemented) to examine the extent to which school personnel adhered to program-recommended targeted intervention procedures, and whether adherence predicted intervention effectiveness.

## Method

### Procedure

Data for the present study came from Finnish primary (*n* = 7), secondary (*n* = 5), and combined (both primary and secondary grades) schools (*n* = 10) implementing the KiVa antibullying program and participating in Challenge project across three academic years (2020–2023). KiVa includes both universal preventive components and procedures for targeted interventions (for a detailed description of the KiVa program, see Kärnä et al., 2013). School personnel responsible for implementing targeted interventions (KiVa teams, typically consisting of three teachers or other personnel) reported each step taken while addressing bullying cases via a mobile application, with one designated team member completing the report for each case. To ensure familiarity with the procedure, team members received a two-hour training in the fall of 2020, which was repeated in fall 2021 for new members. In these sessions, team members were reminded of the definition of bullying (Olweus, 1993) and trained to both follow the intervention procedure when addressing bullying cases and document the intervention steps in the mobile application.

The targeted intervention procedure (as part of *KiVa* program) includes six steps (Fig. 1). In Step 1, the team meets with the victimized student(s) to discuss the situation and provide support. In Step 2, each bullying student is met individually. In Step 3, if multiple perpetrators are involved, a joint meeting with all of them is organized; this step is not relevant when only one perpetrator is identified. In Step 4, the classroom teacher is informed about the bullying and organizes a meeting with a few uninvolved prosocial classmates to encourage them to support the victimized student(s). In Step 5 and Step 6, approximately two weeks after the intervention, two follow-up meetings are recommended: one with the victimized student(s) (Step 5), and another with the bullying student(s), joined by the victim(s) if they were willing to participate (Step 6). In Step 5, the KiVa team asked victimized student(s) whether the bullying had stopped, decreased, remained the same, or increased. In Step 6, the same question was posed to the bullying perpetrator(s) and, if present, also to the victim(s).

In Steps 2 and 3, each team had been instructed to use one of the three approaches: a condemning (affirming that bullying is not acceptable), an empathy-raising (aiming to arouse perpetrators’ empathy for the victim), or a combined approach (both condemning the behavior and raising empathy). The mobile application provided instructions for each step. Comparison between the three approaches was beyond the scope of the present study, as prior research has reported no significant differences in their effectiveness (Laninga-Wijnen et al., 2025).

### Participants

Across the three academic years, the KiVa teams documented 386 bullying cases in the mobile application. After removing test entries, duplicates, cases representing mutual fights or a negative classroom atmosphere rather than bullying, cases created without any further data, and disruptions in the process (e.g., victimized student changing school), 341 documented cases remained. These involved 396 victimized students (209 females, 155 males, 32 with missing gender). The number of perpetrators was unknown in five cases; in the remaining 336 cases, a total of 733 bullying students were involved (97 females, 297 males, 339 with missing gender). Some students – both targets and perpetrators – were involved in more than one case. Because the interventions were conducted and the outcomes were documented at the case level (a “case” involving particular victim(s) and the students bullying them), the present study focused on case-level analyses.

### Measures

#### Adherence to the Intervention Procedure

##### Steps

The KiVa teams reported each intervention step (1–6) through the *KiVappi* mobile application. A team was considered to have *adhered to a step* when it was implemented according to program guidelines, and to have *modified a step* when it was adapted or omitted. Adaptation was defined as doing something else than recommended for the step, and omission as not implementing the step. Reporting was coded as *insufficient* when documentation for the step was missing (i.e., no entries were made by the team) or insufficient to determine adherence; in the majority of cases documentation was missing. Three dummy-coded variables were created: *step adherence*, *step modification*, and *insufficient reporting*.

##### ***Full procedure***

A team was considered to have *adhered to the procedure* when all six steps were implemented as specified. In cases involving multiple perpetrators, Steps 2, 3, and 6 were considered implemented as specified when there was a record of discussion with at least one perpetrator. If any steps were adapted or omitted, the team was considered to have *modified the procedure*. Reporting was coded as *insufficient* when missing or insufficient documentation in one or more steps prevented categorization as adherence or modifications. Three dummy-coded variables were created: *adherence to procedure*, *modifications to procedure*, and *insufficient reporting.*

#### Intervention Effectiveness

During the follow-up meetings school personnel asked the victimized student(s) (Step 5) and bullying students (Step 6) whether the bullying had (1) stopped, (2) decreased, (3) remained the same, or (4) increased. Dummy-coded variables were created for both victim- and bully-reported outcomes: 1 = bullying stopped, 0 = bullying decreased, remained the same, or increased.

#### Control Variables

The proportion of boys and the proportion of students in secondary schools involved in each case were used as case-level control variables.

### Analysis Plan

First, adherence to both the intervention steps and the full procedure was examined. Second, frequencies and percentages of reported change in victimization and bullying perpetration were analyzed across adherence categories. Third, logistic regression analyses were conducted to test whether adherence to the intervention procedure, compared to modifications, predicted changes in bullying behaviors. In the first set of analyses, *modifications to procedure* included both confirmed procedural modifications and cases with insufficient reporting, as insufficient reporting may be indicative of underlying modifications. Separate models were estimated for victimization (Model 1a) and bullying perpetration (Model 2a). A second set of logistic regression analyses excluding cases with insufficient reporting was conducted as a sensitivity analysis (Models 1b and 2b). Lastly, the effects of two types of modifications – adaptations and omissions – were explored relative to full adherence (Models 1c and 2c). All models controlled for school level and the gender proportions of victimized and bullying students. The first and second analyses were conducted with SPSS version 29.0.2.0 (IBM Corp,  2023), and the third with *M*plus 8.8 (Muthén & Muthén, 1998–2022) using Robust Maximum Likelihood Estimation (MLR), with missing data handled through Full Information Maximum Likelihood Estimation (FIML).

## Results

### Descriptive Statistics

Table 1 presents the proportion of cases across adherence categories for each intervention step, based on the KiVa team’s implementation of the procedure. Of the 341 bullying cases, all six intervention steps were implemented according to procedure in only 37 cases (10.9%), whereas in 117 cases (34.3%) the teams had modified the procedure by adapting or omitting steps; in the remaining 187 cases (54.8%), reporting of the steps was insufficient to determine adherence to the procedure. Among the six intervention steps, Step 1 was most frequently adhered to, with no modifications, followed by Step 2, which had one omission. Step 3 was adhered to in slightly less than half of the cases and had the highest modification rate, of which 19.7% were omissions and 80.3% adaptations. Although intended as a group meeting only with the bullying students, the most common adaption was to include the victimized student; in cases with a single perpetrator, this meant a joint meeting with both the victim and the perpetrator. Other adaptations included, for example, involving parents/guardians and/or other school personnel (e.g., the principal) in the meeting or discussing the situation with the whole class. Step 4 had the lowest adherence and the second-highest modification rate (69.2% omissions, 30.8% adaptations). Adaptations included actions such as the classroom teacher discussing the situation with the victimized and/or bullying students, with the whole class, or separately with the boys or girls in the class. Steps 5 and 6 were adhered to in slightly more than half of the cases, with seven modifications in Step 5 (all omissions) and none in Step 6.

**Table 1:** Adherence to targeted intervention procedure

	Step 1		Step 2		Step 3		Step 4		Step 5		Step 6		All steps	
	*n*	*%*	*n*	*%*	*n*	*%*	*n*	*%*	*n*	*%*	*n*	*%*	*n*	*%*
Adherence	336	98.5	299	87.7	155	45.5	57	16.7	195	57.2	193	56.6	37	10.9
Modifications	0	0.0	1	0.3	71	20.8	65	19.1	7	2.1	0	0.0	117	34.3
Insufficient reporting	5	1.5	41	12.0	115	33.7	219	64.2	139	40.8	148	43.4	187	54.8

*N* = 341 bullying cases

For the descriptive analysis of change in victimization and bullying perpetration across adherence categories, cases with missing outcome data were excluded. This resulted in 195 cases for victimization, involving 235 victimized and 405 bullying students, and 189 cases for perpetration, involving 223 victimized and 388 bullying students. Overall, the interventions conducted by the KiVa teams were quite effective: according to victimized students’ reports, victimization had stopped in 72% of the cases, and bullying perpetrators reported that they had stopped bullying in 79% of the cases (see Table 3). However, the intervention effectiveness varied across adherence categories (Table 2). When the KiVa teams adhered to the procedure, victimized students reported that victimization had stopped in 91.9% of cases, compared to 71.7% when the procedure was modified and 62.1% when adherence could not be determined due to insufficient reporting. A similar pattern was observed for perpetrator-reported outcome.

**Table 2:** Reported changes in victimization and perpetration after targeted interventions by adherence to procedure

	Stopped		Decreased		Remained the same		Increased	
Variable	*n*	%	*n*	%	*n*	%	*n*	%
Victimized students								
Adherence to procedure	34	91.9	2	5.4	1	2.7	0	0.0
Modifications to procedure	66	71.7	19	20.7	6	6.5	1	1.1
Insufficient reporting	41	62.1	17	25.8	7	10.6	1	1.5
Bullying students								
Adherence to procedure	34	91.9	2	5.4	1	2.7	0	0.0
Modifications to procedure	76	79.2	15	15.6	5	5.2	0	0.0
Insufficient reporting	39	69.6	13	23.2	4	7.1	0	0.0

*n* for victimized students = 195 bullying cases. *n* for bullying students = 189 bullying cases

### Intervention Effectiveness

#### Victim-reported intervention effectiveness

Correlations and descriptive statistics for the variables included in logistic regression models are presented in Table 3. Model 1a tested the effect of adherence to the intervention procedure on victim-reported intervention effectiveness (Table 4). When the procedure was followed, victimized students were significantly more likely to report that the victimization had stopped than when modifications were made (*OR* = 6.00, *p* =.007), indicating that adherence substantially increased the odds of victimization stopping. The sensitivity analysis yielded a similar result (Model 1b; *OR* = 4.95, *p* =.019).

**Table 3:** Correlations and descriptive statistics of variables included in regression analyses

Variable	1	2	3	4	5	6
1. Victim-reported intervention effectiveness	-					
2. Bully-reported intervention effectiveness	0.74***	-				
3. % Secondary school students*	0.11	0.14*	-			
4. Victimized students: % boy*	0.11*	−0.03	−0.06	-		
5. Bullying students: % boy*	0.12*	0.12*	0.12*	0.48***	-	
6. Adherence to procedure	0.18**	0.12*	−0.03	−0.03	0.07	-
*M*	0.72	0.79	0.48	0.47	0.76	0.11
*SD*	0.45	0.41	0.48	0.50	0.41	0.31

*N* = 341. Correlations coefficients between binary variables are phi coefficients. *The variable represents the percentage of secondary school students/boys involved in the bullying case

***p <.001. **p <.01. *p <.05

**Table 4:** Regression analysis predicting intervention effectiveness

	Victimized students						Bullying students					
Predictor	*b*	*SE*	*OR*	95% *CI*	*Cohen’s d*	*p*	*b*	*SE*	*OR*	95% *CI*	*Cohen’s d*	*p*
	Model 1a						Model 2a					
% Secondary school students*	0.60	0.37	1.83	[0.88, 3.78]	0.33	.104	0.48	0.43	1.62	[0.70, 3.73]	0.27	.259
Victimized students: % boys*	0.70	0.43	2.02	[0.87, 4.67]	0.39	.100	−0.42	0.50	0.65	[0.25, 1.74]	−0.23	.394
Bullying students: % boys*	0.10	0.55	1.10	[0.38, 3.21]	0.05	.862	0.78	0.62	2.17	[0.65, 7.29]	0.43	.210
Adherence to procedure	1.79	0.66	6.00	[1.65, 21.82]	0.99	.007	1.23	0.65	3.41	[0.96, 12.14]	0.68	.058
R^2^	.133	0.07				.057	.088	0.06				.136
	Model 1b						Model 2b					
% Secondary school students*	0.41	0.50	1.50	[0.57, 3.97]	0.22	.413	0.63	0.56	1.87	[0.63, 5.58]	0.35	.262
Victimized students: % boys*	0.69	0.60	2.00	[0.62, 6.44]	0.38	.246	0.05	0.67	1.05	[0.28, 3.92]	0.03	.945
Bullying students: % boys*	0.01	0.74	1.01	[0.24, 4.31]	0.01	.989	0.40	0.83	1.49	[0.29, 7.57]	0.22	.630
Adherence to procedure	1.60	0.68	4.95	[1.29, 18.91]	0.88	.019	1.06	0.68	2.90	[0.77, 10.88]	0.59	.115
R^2^	.150	0.10				.144	.096	0.08				.251
	Model 1c						Model 2c					
% Secondary school students*	0.18	0.61	1.19	[0.36, 3.95]	0.10	.775	−0.14	0.69	0.87	[0.23, 3.37]	−0.07	.845
Victimized students: % boys*	0.69	0.70	1.99	[0.50, 7.91]	0.38	.329	−0.09	0.87	0.92	[0.17, 5.05]	−0.05	.921
Bullying students: % boys*	−0.02	0.86	0.98	[0.18, 5.28]	−0.01	.985	0.71	0.96	2.03	[0.31, 13.46]	0.39	.461
Adaptations to procedure	−1.16	0.82	0.31	[0.06, 1.57]	−0.64	.157	−0.36	0.92	0.70	[0.12, 4.24]	−0.20	.698
Omissions from procedure	−1.76	0.75	0.17	[0.04, 0.75]	−0.97	.019	−1.14	0.74	0.32	[0.08, 1.37]	−0.63	.125
R^2^	.174	0.13				.169	.099	0.10				.341

*n* = 341 (Model 1a and 2a), 154 (Model 1b and 2b), and 96 cases (Model 1c and 2c). In Models 1a/1b and 2a/2b the reference category is modifications to procedure. For Model 1a and 2a, this includes both confirmed modifications and cases with insufficient reporting; for Model 1b and 2b, only confirmed modifications. In Model 1c and 2c the reference category is adherence to procedure. *The variable represents the percentage of secondary school students/boys involved in the bullying case

As an additional exploratory analysis, the effects of two types of modifications – adaptations and omissions – were compared to full adherence (Model 1c). Analyses were restricted to cases involving only adaptations or only omissions alongside full adherence. When steps were omitted, victimized students were significantly less likely to report that the victimization had stopped compared to cases of full adherence (*OR* = 0.17, *p* =.019), corresponding to about 5.9 times lower odds. In contrast, when steps were adapted, the likelihood of reporting that the victimization had stopped did not differ significantly from full adherence (*OR* = 0.31, *p* =.157), although the association was in the same direction as in omissions.

#### Bully-reported intervention effectiveness

Model 2a included the same predictors as Model 1a but relied on bully-reported outcome (Table 4). Results were consistent with victim reports, although attenuated, and they did not reach statistical significance (*OR* = 3.41, *p* =.058), suggesting a tendency for adherence to increase the odds of perpetration ceasing. The sensitivity analysis also did not show a significant association between adherence to procedure and the outcome (*OR* = 2.90, *p* =.115). Exploratory analyses (Model 2c) examined the effects of adaptations and omissions relative to full adherence. None of the associations were significant (adaptations; *OR* = 0.70, *p* =.698; omissions *OR* = 0.32, *p* =.125).

## Discussion

The KiVa teams’ adherence to the evidence-based targeted intervention procedure varied considerably across the intervention steps, and adherence to the procedure was low, with all six steps implemented as specified in the protocol in only 10.9% of the cases. Thus, in most instances, KiVa teams either made modifications to the procedure by adapting or omitting steps (34.3%) or provided insufficient reporting to determine adherence to the procedure (54.8%). Although the interventions were overall quite effective in stopping victimization and bullying – a finding consistent with previous targeted intervention studies (e.g., Garandeau et al., 2014; Johander et al., 2024) – their effectiveness was found to depend on the adherence to intervention guidelines: the interventions were more successful when teams had adhered to the intervention procedure rather than made modifications to it. This was especially evident in victimized students’ reports. The interventions were least effective when steps were omitted, whereas adaptations did not significantly reduce effectiveness compared to full adherence, though the trend was similar as with omissions. Again, this effect was evident in victim reports, with a similar non-significant trend also appearing in perpetrators’ reports. In line with previous research on implementation fidelity (Durlak & DuPre, 2008), these findings provide further evidence for the importance of adhering to evidence-based protocols for the effectiveness of targeted interventions in stopping bullying (Johander et al., 2021). In addition, they suggest that omitting intervention steps may be more detrimental to the effectiveness of targeted bullying interventions than adaptations.

The study has several strengths, notably the examination of the full targeted intervention procedure, the focus on two types of modifications (adaptations and omissions), and the use of ecological momentary assessments. However, it also has limitations. First, due to the large amount of missing data on implementation, adherence to the procedure could only be determined in about half of the cases. Second, despite the association between adherence and intervention outcomes, the relatively small sample size limits the strength of the conclusions and the generalizability of the findings. Third, students’ outcome reports might be affected by social desirability bias. Fourth, the small number of adaptation-only cases precluded a more fine-grained classification of modifications. Future studies allowing more detailed analyses are needed to further improve understanding of how different types of modifications beyond the distinction between omissions and adaptations (e.g., addition of elements or the integration of elements from another intervention; Stirman et al., 2013) relate to intervention effectiveness. Finally, we did not collect information on why KiVa teams made modifications to the procedure. In a few cases, however, reasons were reported – for instance, omitting Step 4 because it was considered unnecessary or because the classroom teacher felt that no one in the class could directly support the victimized student. Some adaptations, in turn, may have been made in response to particularly challenging cases, in which their lower effectiveness could reflect the severity of the bullying rather than the ineffectiveness of the adaptations themselves. Modifications may also occur due to lack of time or resources (Herkama et al., 2022). Thus, future studies should investigate the reasons for modifications to further clarify their effects on intervention effectiveness.

## Conclusions

The KiVa teams’ adherence to program-recommended targeted intervention procedure varied considerably across the intervention steps, and adherence to the full procedure was low. While the interventions were generally effective in stopping victimization and bullying perpetration, their success depended on adherence to the recommended procedure. Interventions were most effective, when the full procedure was followed, and omissions appeared more detrimental to the effectiveness than adaptations.

The results of this study do not suggest that there is only one correct targeted intervention procedure that must be strictly followed to effectively stop bullying. Rather, they suggest that following evidence-based guidelines shown to be successful (e.g., Garandeau et al., 2014) leads, on average, to better outcomes than merely doing something. Following the recommended procedure may also be an indication of school personnel taking bullying seriously and striving to intervene effectively. Taking multiple steps (rather than, for example, only talking once to the bullying students) may itself already convey to students that adults are committed to addressing bullying. Besides such an attitude, other aspects of implementation quality (see Tolmatcheff et al., 2024), such as clarity of communication, and teacher-student interaction, are also likely to affect intervention effectiveness.

## Data Availability

The fully anonymized dataset is available from the last author upon reasonable request.
